# Contact, high-resolution spatial diffuse reflectance imaging system for skin condition diagnosis: a first-in-human clinical trial

**DOI:** 10.1117/1.JBO.26.1.012706

**Published:** 2021-01-29

**Authors:** Anne Koenig, Nils Petitdidier, Henri Grateau, Samarmar Characoun, Abdallah Ghaith, Samuel Verges, Stéphane Doutreleau, Sadok Gharbi, Rémi Gerbelot, Sylvain Gioux, Jean-Marc Dinten

**Affiliations:** a Université Grenoble Alpes, France—CEA, LETI, MINATEC Campus, Grenoble, France; b Université de Strasbourg, Strasbourg, France; c Université Grenoble Alpes, CHU Grenoble Alpes, INSERM, Hôpital Sud, Laboratoire HP2 (U 1042), Avenue Kimberley, Echirolles, France; d Télécom Physique Strasbourg, Laboratoire ICube, Illkirch, France

**Keywords:** skin characterization, optical properties, diffuse reflectance, multipixel sensor, contact imaging, tissue oxygen saturation, wearable device

## Abstract

**Significance:** Oxygenation is one of the skin tissue physiological properties to follow for patient care management. Furthermore, long-term monitoring of such parameters is needed at the patient bed as well as outside the hospital. Diffuse reflectance spectroscopy has been widely used for this purpose.

**Aim:** The aim of the study is to propose a low-cost system for the long-term measurement of skin physiological parameters in contact.

**Approach:** We have developed a low-cost, wearable, CMOS-based device. We propose an original method for processing diffuse reflectance data to calculate the tissue oxygen saturation (${\mathrm{StO}}_{2}$).

**Results:** We tested the device for the assessment of tissue oxygenation during a first-in-human clinical trial that took place at the Grenoble University Hospital France.

**Conclusions:** The results of this clinical trial show a good accordance between our sensor and commercial devices used a reference.

## 1 Introduction

Knowledge of tissue properties such as oxygen saturation are important in patient care management in a great number of pathologies. Long-term monitoring of such parameters is currently needed at the patient bed as well as outside the hospital. The development of tools capable of monitoring physiological parameters could be of great interest also for applications *in situ* at the hospital such as tissue oxygenation monitoring toward postoperative assessment of superficial flaps viability.^1^ In addition, the use of these instruments outside the hospital will become more and more necessary toward the development of remote medicine of tomorrow. Unfortunately, as of today, few instruments are able to assess the tissue viability continuously, in the long term and at low cost. Near-infrared spectroscopy is one of the available techniques providing relative concentrations of deoxygenated and oxygenated hemoglobin and calculation of an index of tissue oxygenation.^2^ This technique is used in a research setting and in some specialized medical units.^3^

Diffuse reflectance spectroscopy (DRS) has been widely used in the field of biological tissue characterization with various modalities. One of these modalities consists of measuring the spatially resolved diffuse reflectance (srDR), collecting light at multiple distances from the excitation point. The obtained reflectance decay curve is used to determine scattering and absorption properties of the tissue,^4^ which are directly related to tissue content and structure. Existing spatially resolved diffuse reflectance spectroscopy (srDRS) systems usually use fiber-optic probes to collect light reflected from the tissue and transfer it to an optical sensor.^5^ Recently, efforts have been made to miniaturize the technology through the use of custom photodiodes to detect photons at the tissue surface.^6^ As part of the previous work, we have developed a wearable device based on an innovative architecture for srDRS using a commercially available complementary metal oxide semiconductor (CMOS) sensor placed in contact with the tissue.^7^ In this paper, we present results obtained using this CMOS-DRS device to monitor the oxygenation of tissue *in vivo* during a first-in-human clinical trial where desaturation was controlled and followed by an ischemia. We first describe the instrument, the data processing methodology, and the protocol of the clinical trial. Then, we detail and compare results obtained *in vivo* using this sensor and commercial reference systems.

## 2 Material and Method

### 2.1 Sensor Design

srDRS is a powerful tool for quantitative tissue analysis using cost-effective and compact systems. As a way to overcome the shortcomings of current systems, usually using fibered probes, we proposed an innovative srDRS architecture to achieve contact, high-resolution imaging of the diffuse reflectance from tissue.^7^ While previous studies necessitated the development of custom photodiode arrays,^6^ we proposed to implement a system using low-cost, commercially available components including a CMOS image sensor and light-emitting diodes. The limitations of this approach concerning the access to short source detector separation (SDS) were identified, and an instrumental solution to circumvent these limitations was proposed. This srDRS sensor architecture herein referred to as CMOS-DRS is based on the insertion of a fiber-optic plate (FOP) between the sensor and tissue.^8^

**Fig. 1:**
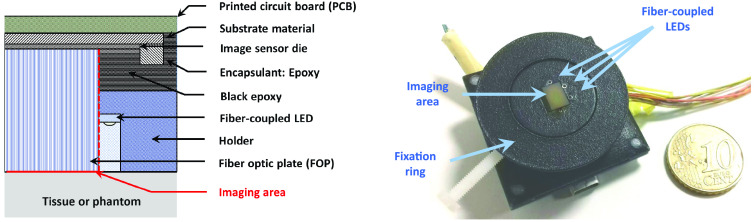
Layout and photograph of the CMOS-based srDRS prototype.

The CMOS-DRS system is shown in Fig. 1. The custom FOP with 6-μm unit fiber core diameter (OS-ST, SZPhoton) is inserted between the sensor [CMOS sensor (UI-1492 IDS imaging Inc.) with 6.413  mm×4.589  mm area and 1.67  μm pixel pitch] and the analyzed medium. The FOP is positioned to ensure alignment of the pixel area borders with the FOP edges. Four fiber-coupled luminescent electronic diodes (LED) (Kingbright Inc.) emitting at λ=515, 611, and 660 nm are placed next to the FOP side and are used as excitation sources. They were chosen because of their small lateral dimension (0.8 mm), which was required to provide access to sufficiently small SDS. The sources and FOP are hosted in a specifically designed packaging module. The packaging was 3-D printed in a biocompatible polyamide (PA2200). A central hole corresponding to the imaging area was left free for insertion of the FOP. The module is mounted on the camera using plastic screws at the corners of the printed circuit board. For *in vivo* measurements, a dedicated fixation ring is first attached to the tissue using standard medical adhesive tapes. The device is then inserted through the ring and set with a screw to maintain a constant pressure on the tissue. The outer surface of the packaging was painted in black to limit unwanted reflections at the tissue-exterior interface.

In summary, the CMOS-DRS system lighting module has the characteristics listed in Table 1.

**Table 1:** Lighting module characteristics.

Color	Wavelength (nm)	SDS min (μm)	SDS max (mm)
Green	515	760	7
Orange	611	820	7
Red	660	690	7

The sensor was connected via USB connection to a computer (Dell Latitude E6530, 64 bits, 4 Go RAM, Intel^®^ CoreTM i3). The acquisition was controlled using a dedicated LabVIEW interface (National Instruments Inc., Austin, Texas).

### 2.2 Image Acquisition Sequence

Diffuse reflectance images were stored and processed on the computer. All imaging processing steps were performed using Matlab (Mathworks Inc., Natick, Massachusetts). Raw reflectance images and dark noise images (background subtraction) were first recorded at multiple exposure times. Typically, for the clinical trial experiments, we used two exposure times. The obtained images were recombined to expand the sensor dynamic (dynamic expansion). Then, the geometry of our collecting light system using the CMOS camera was exploited by ring averaging. The pixel values were averaged over concentric annular areas surrounding the illumination center to extract the radial profile of raw diffuse reflectance.^7^

The LEDs were lit in the following order: orange, red, green, with the first exposure time for the first image acquisition, then with the second exposure time (equal to the first exposure time × 8) for the second image acquisition. The exposure times ranges are presented in Table 2.

**Table 2:** Exposure times.

Color	Time 1 (ms)	Time 2 (ms)
Green	1.3<t1<7.28	10.52<t2<26.08
Orange	1.15<t1<3.26	9.5<t2<76.1
Red	7.5<t1<18.04	60.3<t2<144.3

### 2.3 Method for Data Processing

**Fig. 2:**
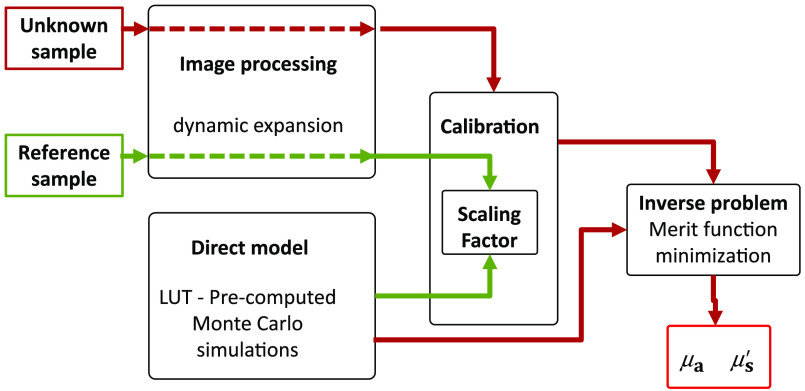
Flowchart of the data processing steps for optical properties quantification.

In the srDR technique, the measured diffuse reflectance decay curves are exploited to determine the scattering and absorption properties of the analyzed tissue.^7^ Data processing was conducted according to the flowchart shown in Fig. 2.

A Monte Carlo (MC)^9^ algorithm was used to simulate the propagation of photons in tissue. The source diameter and numerical aperture (NA) were accounted for in the computation. The theoretical reflectance profiles calculated for various $\mu_{a}$ and ${\mu_{s}}^{\prime }$ were stored in a look-up table. Following an approach described in previous studies,^10^ measured and theoretical reflectance profiles were matched through merit function minimization to extract the optical properties of the tissue. Performances of the designed prototype and of the method to extract optical properties (i.e., $\mu_{a}$ and ${\mu_{s}}^{\prime }$ coefficients) were evaluated with tissue-simulating phantoms.^7^

### 2.4 Calibration Phantoms

To calibrate the system, diffuse reflectance measurements were performed on a phantom having known optical properties. We used a range of homemade solid tissue-simulating phantoms manufactured in polydimethylsiloxane (PDMS). Ethanol-dissolved nigrosin (Ng-EtOH) and titanium dioxide (${\mathrm{TiO}}_{2}$) particles were added in the phantom mixture to generate absorption and scattering.^8^^,^^11^

Phantoms optical properties at 665 and 860 nm were characterized through spatial frequency domain imaging (SFDI). The obtained $\mu_{a}$ and ${\mu_{s}}^{\prime }$ optical coefficients were then extrapolated at 511, 615, and 660 nm. Several authors have measured scattering spectra of ${\mathrm{TiO}}_{2}$-based PDMS phantoms, showing a nearly linear behavior in the 500 to 900 nm range.^12^^–^^14^ Therefore, linear extrapolation using the values of ${\mu_{s}}^{\prime }$ available at 665 and 860 nm was used to calculate the reduced scattering coefficient of the calibration phantom at our wavelengths of interest, i.e., 511, 615, and 660 nm. Extrapolation of $\mu_{a}$ coefficients was based on spectra measured with an srDRS fiber-optic probe previously developed at CEA laboratory.^5^

The calibration phantom measurement was used to yield a scaling factor through comparison with the theoretical data (box “calibration” of the flowchart Fig. 2). The scaling factor was applied to unknown object (phantom or tissue) measurements to convert raw diffuse reflectance profiles into absolute unit.

During this study, we used two calibration phantoms with properties presented in Table 3. Depending on subjects’ phototype, we used one or the other of these phantoms. Several authors pointed out that errors in optical properties determination should be minimal when the scattering coefficients of the calibration phantom and the analyzed medium are the same.^15^ In the case of using multiple calibration phantoms, the work done by Sorgato et al.^16^ could help with choosing the best adapted.

**Table 3:** Calibration phantoms optical properties at 665 nm.

Name	Nigrosin concentration (mg/g)	${\mathrm{TiO}}_{2}$ concentration (mg/g)	$\mu_{a}$ @ 665 nm (${\mathrm{cm}}^{-1}$)	${\mu_{s}}^{\prime }$ @ 665 nm (${\mathrm{cm}}^{-1}$)
B1	0.08	3	0.52	19.4
B2	0.239	3	0.99	18.2

### 2.5 Derivation of the Tissue Oxygen Saturation

We have implemented two original methods to extract tissue oxygen saturation from optical coefficients estimates. The first method hereafter referenced as “method A” uses data measured at two wavelengths only and requires a secondary reference system for calibration. To avoid the need of a reference system, a second approach using a three-wavelengths acquisition and processing scheme was investigated, it is referenced as “method B.”

#### 2.5.1 Method A

The measured wavelength-dependent absorption coefficient was exploited to determine the relative concentration of oxy- and deoxygenated blood in tissue, which is represented by the tissue oxygen saturation parameter (${\mathrm{StO}}_{2}$). In method A, images recorded at wavelengths $\lambda_{1}=660\;\mathrm{nm}$ and $\lambda_{2}=611\;\mathrm{nm}$ were used.

Using modeling based on volume fractions proposed by Jacques,^17^ absorption in skin was modeled as

$$\mu_{a}(\lambda ,t)=B(t)*\{{\mathrm{StO}}_{2}(t)*\mu_{a}^{{\mathrm{HbO}}_{2}}(\lambda )+[1-{\mathrm{StO}}_{2}(t)]{*\mu }_{a}^{Hb}(\lambda )\}+[1-B(t)]{*\mu }_{a}^{\text{other}}(\lambda ,t),$$ (1)

where $\mu_{a}(\lambda ,t)$ is the absorption coefficient measured by the CMOS-DRS device over time t (${\mathrm{cm}}^{-1}$), B(t) stands for the blood volume fraction in tissue as defined by Jacques^17^ or Meglinski and Matcher, ^18^$\;{\mathrm{StO}}_{2}(t)$ represents the tissue saturation, $\mu_{a}^{{\mathrm{HbO}}_{2}}(\lambda )$ and $\mu_{a}^{\mathrm{Hb}}(\lambda )$ are the tabulated spectra of oxygenated and deoxygenated blood absorption^19^ (${\mathrm{cm}}^{-1}$), $\mu_{a}^{\text{other}}(\lambda ,t)$ gathers the contributions of “other” chemical species to the skin absorption (melanin, bilirubin,…) (${\mathrm{cm}}^{-1}$).

Meglinski and Matcher proposed an analytic formula to express a “mean” $\mu_{a}^{\text{other}}$ as a function of wavelength:^18^

$$\mu_{a}^{\text{other}}(\lambda )=7.84*10^{8}*\lambda^{-3.255},$$ (2)

where λ is the wavelength expressed in nanometers.

To account for intersubject variations in background absorption, especially related to varying melanin content, we introduced an additional calibration factor x(t), the exponential shape of absorption decay with wavelength of Eq. (2) being conserved, $\mu_{a}^{\text{other}}(\lambda ,t)$ thus writes:

$$\mu_{a}^{\text{other}}(\lambda ,t)=x(t)*\;7.84*10^{8}*\lambda^{-3.255},$$ (3)

where x(t) is characteristic of each subject.

In order to calculate the ${\mathrm{StO}}_{2}$, we solve a system of two equations with two wavelengths:

$$\left\{ \begin{array}{c} \mu_{a}(\lambda_{1},t)=B(t)*\{{\mathrm{StO}}_{2}(t){*\mu }_{a}^{{\mathrm{HbO}}_{2}}(\lambda_{1})+[1-{\mathrm{StO}}_{2}(t)]{*\mu }_{a}^{Hb}(\lambda_{1})\}+[1-B(t)]*\mu_{a}^{\text{other}}(\lambda_{1},t)\\ \mu_{a}(\lambda_{2},t)=B(t)*\{{\mathrm{StO}}_{2}(t){*\mu }_{a}^{{\mathrm{HbO}}_{2}}(\lambda_{2})+[1-{\mathrm{StO}}_{2}(t)]{*\mu }_{a}^{\mathrm{Hb}}(\lambda_{2})\}+[1-B(t)]*\mu_{a}^{\text{other}}(\lambda_{2},t) \end{array}.\right.$$ (4)

As described in Sec. 2.2, diffuse reflectance images for wavelengths $\lambda_{1}$ and $\lambda_{2}$ were recorded sequentially during *in vivo* experiments. Prior to ${\mathrm{StO}}_{2}$ calculation, $\mu_{a}$ measurements for $\lambda_{2}$ were therefore readjusted to the acquisition times of $\lambda_{1}$. The resolution of this system gives the following relations:

With:

$$\left\{ \begin{array}{l} C_{1i}=\mu_{a}(t,\lambda_{i})-\mu_{a}^{\text{other}}(\lambda_{i},t)\\ C_{2i}=\mu_{a}^{\mathrm{Hb}}(\lambda_{i})-\mu_{a}^{\text{other}}(\lambda_{i},t)\\ C_{3i}=\mu_{a}^{{\mathrm{HbO}}_{2}}(\lambda_{i})-\mu_{a}^{\mathrm{Hb}}(\lambda_{i}) \end{array},\right.$$

$$\left\{ \begin{array}{l} {\mathrm{StO}}_{2}(t)=\frac{C_{11}C_{22}-C_{21}C_{12}}{C_{31}C_{12}-C_{32}C_{11}}\\ B(t)=\frac{C_{11}(C_{31}C_{12}-C_{32}C_{11})}{C_{31}(C_{11}C_{22}-C_{21}C_{12})+C_{21}(C_{31}C_{12}-C_{32}C_{11})} \end{array}.\right.$$ (5)

Calibration of the system was based on the comparison of readings recorded during the initial stable period of the protocol (ambient air step as mentioned in Sec. 2.6) by the CMOS-DRS device and a reference ${\mathrm{StO}}_{2}$ sensor (PortaLite, Artinis Medical Systems, The Netherlands, referred hereafter as “Artinis”^20^). The average ${\mathrm{StO}}_{2}$ value provided by the Artinis noted $S_{\mathrm{bsl}}$, and $\mu_{a}$ value extracted by the CMOS-DRS system noted $\mu_{\mathrm{absl}}$ yield the calibration factor x specific to each subject. Note that with this method A, we do not take the variation of x over time into account but consider an average value of x over a stable time interval:

$$x=\frac{\left\{S_{\mathrm{bsl}}*[\mu_{a\;\mathrm{bsl}}(\lambda_{2})*C_{31}-\mu_{a\;\mathrm{bsl}}(\lambda_{1})*C_{32}]+\mu_{a\;\mathrm{bsl}}(\lambda_{2}){*\mu }_{a}^{\mathrm{Hb}}(\lambda_{1})-\mu_{a\;\mathrm{bsl}}(\lambda_{1}){*\mu }_{a}^{\mathrm{Hb}}(\lambda_{2})\right\}}{7.84*10^{8}*\{\lambda_{1}^{-3.255}*[\mu_{a\;\mathrm{bsl}}(\lambda_{2})-\mu_{a}^{\mathrm{Hb}}(\lambda_{2})+S_{\mathrm{bsl}}*C_{32}]-\lambda_{2}^{-3.255}*[\mu_{a\;\mathrm{bsl}}(\lambda_{1})-\mu_{a}^{\mathrm{Hb}}(\lambda_{1})+S_{\mathrm{bsl}}*C_{31}]\}}.$$ (6)

#### 2.5.2 Method B

As a second approach, we used the absorption coefficient measured at a third wavelength (511 nm) to determine the calibration factor x(t), thereby avoiding the use of a reference sensor.

Using Eqs. (1) and (3), and with the following notation, Eq. (1) leads to Eq. (7):

$$L(\lambda )=7.84*10^{8}*\lambda^{-3.255}\;(\lambda \text{ in }\mathrm{nm})$$

$$\mu_{a}(\lambda ,t)=B(t){*\mathrm{StO}}_{2}(t)[\mu_{a}^{{\mathrm{HbO}}_{2}}(\lambda )-\mu_{a}^{\mathrm{Hb}}(\lambda )]+B(t){*\mu }_{a}^{\mathrm{Hb}}(\lambda )+[1-B(t)]*x(t)*L(\lambda ).$$ (7)

With:

$$\begin{array}{lll} \begin{array}{c} C_{11}=\mu_{a}(\lambda_{1},t)L(\lambda_{2})\\ -\mu_{a}(\lambda_{2},t)L(\lambda_{1}) \end{array} & \begin{array}{c} C_{12}=\mu_{a}(\lambda_{2},t)L(\lambda_{3})\\ -\mu_{a}(\lambda_{3},t)L(\lambda_{2}) \end{array} & \begin{array}{c} C_{13}=\mu_{a}(\lambda_{3},t)L(\lambda_{1})\\ -\mu_{a}(\lambda_{1},t)L(\lambda_{3}) \end{array}\\ \begin{array}{c} C_{21}=\mu_{a}^{\mathrm{Hb}}(\lambda_{1})L(\lambda_{2})\\ -\mu_{a}^{\mathrm{Hb}}(\lambda_{2})L(\lambda_{1}) \end{array} & \begin{array}{c} C_{21}=\mu_{a}^{\mathrm{Hb}}(\lambda_{2})L(\lambda_{3})\\ -\mu_{a}^{\mathrm{Hb}}(\lambda_{3})L(\lambda_{2}) \end{array} & \begin{array}{c} C_{21}=\mu_{a}^{\mathrm{Hb}}(\lambda_{3})L(\lambda_{1})\\ -\mu_{a}^{\mathrm{Hb}}(\lambda_{1})L(\lambda_{3}) \end{array}\\ \begin{array}{c} C_{31}=\mu_{a}^{{\mathrm{HbO}}_{2}}(\lambda_{1})-\mu_{a}^{\mathrm{Hb}}(\lambda_{1}) \end{array} & \begin{array}{c} C_{32}=\mu_{a}^{{\mathrm{HbO}}_{2}}(\lambda_{2})-\mu_{a}^{\mathrm{Hb}}(\lambda_{2}) \end{array} & \begin{array}{c} C_{33}=\mu_{a}^{{\mathrm{HbO}}_{2}}(\lambda_{3})-\mu_{a}^{\mathrm{Hb}}(\lambda_{3}) \end{array}\\ \begin{array}{c} C_{41}=\mu_{a}^{\mathrm{Hb}}(\lambda_{1})\mu_{a}(\lambda_{2},t)\\ -\mu_{a}^{\mathrm{Hb}}(\lambda_{2})\mu_{a}(\lambda_{1},t) \end{array} & \begin{array}{c} C_{41}=\mu_{a}^{\mathrm{Hb}}(\lambda_{2})\mu_{a}(\lambda_{3},t)\\ -\mu_{a}^{\mathrm{Hb}}(\lambda_{3})\mu_{a}(\lambda_{2},t) \end{array} & \begin{array}{c} C_{41}=\mu_{a}^{\mathrm{Hb}}(\lambda_{3})\mu_{a}(\lambda_{1},t)\\ -\mu_{a}^{\mathrm{Hb}}(\lambda_{1})\mu_{a}(\lambda_{3},t) \end{array} \end{array}$$

The resolution of this new system gives the following relations for ${\mathrm{StO}}_{2}$ calculation:

$$\left\{ \begin{array}{l} B(t)=\frac{C_{31}C_{12}+C_{32}C_{13}+C_{33}C_{11}}{C_{31}C_{22}+C_{32}C_{23}+C_{33}C_{21}}\\ {\mathrm{StO}}_{2}(t)=\frac{\mu_{1}C_{22}+\mu_{2}C_{23}+\mu_{3}C_{21}}{C_{31}C_{12}+C_{32}C_{13}+C_{33}C_{11}}\\ x(t)=\frac{C_{31}C_{42}+C_{32}C_{43}+C_{33}C_{41}}{C_{31}(C_{22}-C_{12})+C_{32}(C_{23}-C_{13})+C_{33}(C_{21}-C_{11})} \end{array}.\right.$$ (8)

### 2.6 First-in-Human Clinical Trial

We planned a clinical trial that began at the beginning of June 2019. This trial aimed at testing two sensors that will eventually be on the same platform to measure two physiological parameters: partial pressure of carbon dioxide (${\mathrm{PtCO}}_{2}$) and ${\mathrm{StO}}_{2}$.^21^ The clinical trial has been performed in collaboration with INSERM & CHU Grenoble University Hospital. It was planned to acquire data on a panel of 20 healthy volunteers [male and female, nonsmoker (attested by exhaled CO<8  ppb), free from any disease and medication except contraceptives, IMC (body mass index) <27] aged from 20 to 50 years old. The subjects’ phototype varied from light to dark skin. Power assessment was based on a minimum expected difference in tissue saturation index of 2% between the two methods. With an α (type I error rate) level of 5% and power of 80%, 15 subjects were required. To take account of potential technical issues, 20 subjects were included in the trial.

Our institutional review board (CPP Grenoble Sud Est III) approved this study (CTCO, 38RC18.176) and all subjects included signed a written informed consent. Experiments were performed according to the Declaration of Helsinki (clinical trial registration: NCT03992651).

**Fig. 3:**
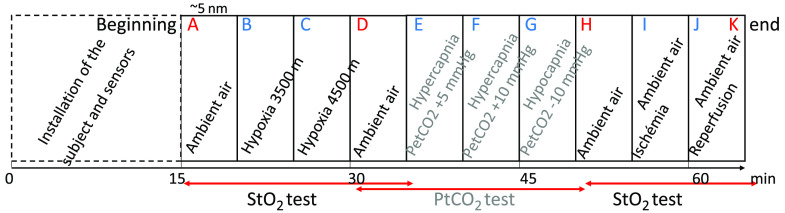
Protocol steps summary.

The protocol is shown in Fig. 3. For each volunteer, the experiment was divided in several steps (from A to K) corresponding to different levels of hypoxia, hypocapnia, hypercapnia, or ischemia. The subject breathed through a nasobucal mask allowing the inhalation of different gas mixtures controlled by the Altitrainer (ALTITRAINER200^®^, S.M.TEC, Geneva, Switzerland)^22^ system. Hypoxic, hypercapnic, gas mixture are obtained by adding nitrogen, carbon dioxide, to ambient air.

Before and after each volunteer measurements, acquisitions on reference phantoms were performed (not mentioned in Fig. 3).

- Three levels of hypoxia were targeted (steps A, B, C). The first target was ambient air at an altitude of 200 m, the subject breathed normally through the nasobucal mask. Then, the gas mixture was changed to simulate breathing at altitudes of 3500 and 4500 m, this last target corresponding to a pulse oxygen saturation level (${\mathrm{SpO}}_{2}$) of ∼80%. Once reached, the target hypoxic levels were kept during 3 min at least. To reach distinct levels of ${\mathrm{SpO}}_{2}$ for each subject, various levels of inspiratory oxygen fractions (${\mathrm{FiO}}_{2}$) were controlled by the Altitrainer system. It is worth noting that this approach led to some interindividual heterogeneity in ${\mathrm{SpO}}_{2}$ levels, however, allowing us to compare the different devices (CMOS-DRS, ${\mathrm{SpO}}_{2}$, PortaLite) throughout a relatively large spectrum of ${\mathrm{SpO}}_{2}$ levels.
- Ambient air step (D) was done to retrieve normal parameters.
- Three consecutive hypercapnia or hypocapnia were targeted (E, F, G), for the test of the capnometry sensor.^23^
- Ambient air step (H) was done to retrieve normal parameters.
- Ischemia of the right arm (step I) was induced using a cuff inflated at 250 mmHg pressure. This step aimed at evaluating the sensitivity of the device to tissue oxygenation changes, following an approach used in previous studies.^2^ During a last step starting from the pressure cuff release, recording of skin parameters was maintained to monitor the reoxygenation of tissue. Two acquisitions were made, one right after cuff release (J) and the other at the end of the experiment (K).

By restricting the supply of blood to the vessels in the arm, upstream of the sensor, ischemia aims to simulate a quick shortage of oxygen in the forearm. This step of ischemia is the true test of our system to detect flap failure.

**Fig. 4:**
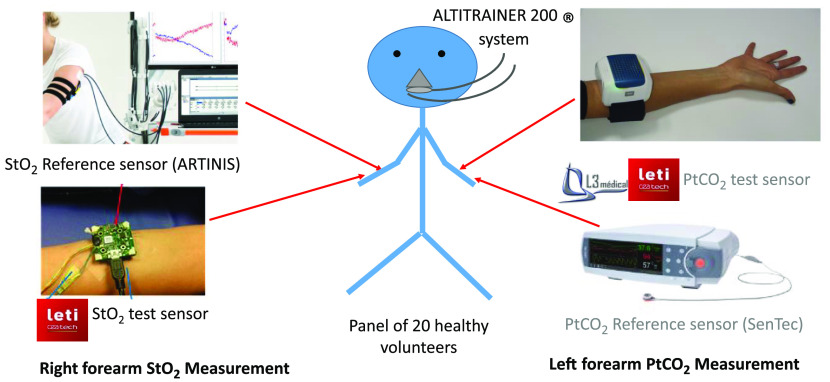
Schematic and photograph of the sensors placement on the volunteer.

**Fig. 5:**
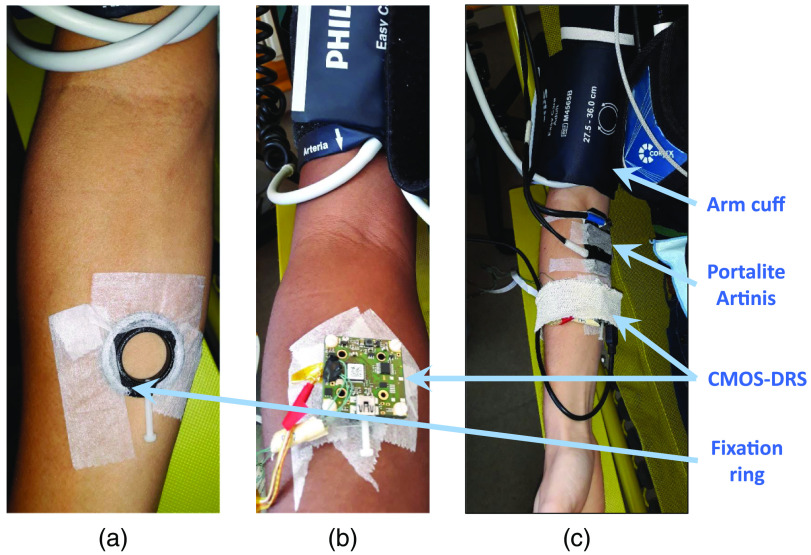
Installation steps of sensors for *in vivo* ${\mathrm{StO}}_{2}$ measurement. (a) Attachment of the CMOS-DRS system was performed using a fixation ring and medical adhesive tapes. (b) The wearable CMOS-DRS system was placed on the inner forearm and away from the Artinis illumination field to avoid parasite light signals. (c) An ischemia of the upper right arm was performed using a cuff inflated at 250-mmHg pressure.

The photographs presented in Figs. 4 and 5 illustrate this experimental protocol. The left arm was dedicated to capnometry sensors while ${\mathrm{StO}}_{2}$ sensors were placed on the right arm.

Continuous monitoring of tissue optical properties was carried out on the volar right forearm using the CMOS-DRS device and an NIRS system simultaneously. The NIRS PortaLite sensor, which is in routine use in our hospital to measure tissue oxygenation in muscles,^24^ was chosen as reference system for this study. Particular attention was paid to the installation of the devices to ensure that optical paths of the two systems would not interfere. Diffuse reflectance acquisitions were carried out for all the protocol duration, including hypo- and hypercapnia. The total duration of the test was about 60 min.

The so-named CAPNO device introduces an innovative noninvasive transcutaneous ${\mathrm{PtCO}}_{2}$ measurement method (or ${\mathrm{TcpCO}}_{2}$). The measurement principle relies on the absorption of infrared radiation by the carbon dioxide released through skin.^25^ The skin surface is heated to 42°C. CAPNO device drives the carbon dioxide emanating from the skin through an optical measurement cell (thermopile and near-infrared source). The amount of radiation received by the thermopile is related to the concentration of transcutaneous carbon dioxide. During the experiment, measurements were compared with a commercially available electrochemical sensor:(SenTec, V-Sign, Switzerland).^26^ This system is composed of a transcutaneous electrochemical ${\mathrm{tcPCO}}_{2}$ sensor and of a reflectance optical ${\mathrm{SpO}}_{2}$ sensor. It allows continuous measurement of ${\mathrm{tcPCO}}_{2}$, pulse oximetry (${\mathrm{SpO}}_{2}$, pulse rate). In addition to ${\mathrm{tcPCO}}_{2}$ reference measurements, ${\mathrm{SpO}}_{2}$ readings were recorded on the left arm using the SenTec sensor. Results concerning CAPNO test are the subject of a separate communication.^23^

## 3 Results

**Fig. 6:**
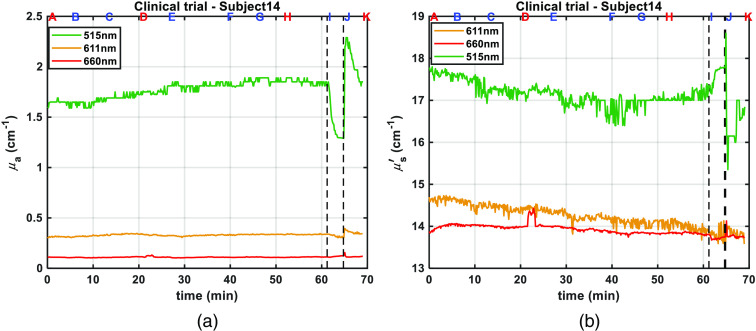
Optical properties: (a) absorption coefficient, (b) reduced scattering coefficient, measured for subject 14 using phantom B2 as reference, at 515 nm (green), 611 nm (orange), and 660 nm (red). Letters on top of figures correspond to protocol steps, and black dashed lines correspond to ischemia steps I and J.

**Fig. 7:**
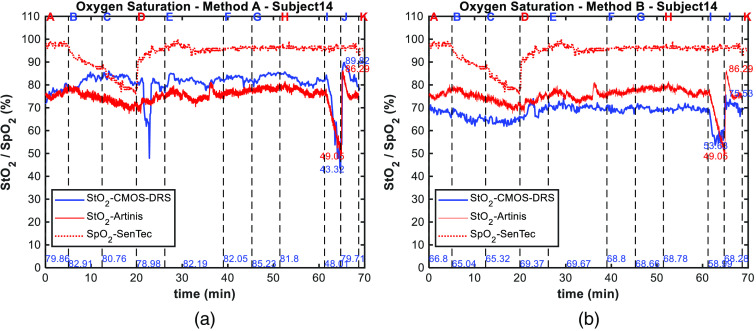
(a) Oxygen saturation readings for subject 14 using method A, (b) using method B. Measurements of the tissue oxygen saturation obtained using the CMOS-DRS (blue line) and Artinis (red line) systems are plotted along with the pulsed oxygen saturation measured by the SenTec sensor (red dotted line). At the bottom of figure are indicated in blue mean CMOS-DRS ${\mathrm{StO}}_{2}$ values for last 30 s of each protocol step, and near the curves min and max of ${\mathrm{StO}}_{2}$ value at ischemia for both sensors (in blue for CMOS-DRS and in red for Artinis). Vertical black dashed lines and letters on top correspond to protocol steps.

**Fig. 8:**
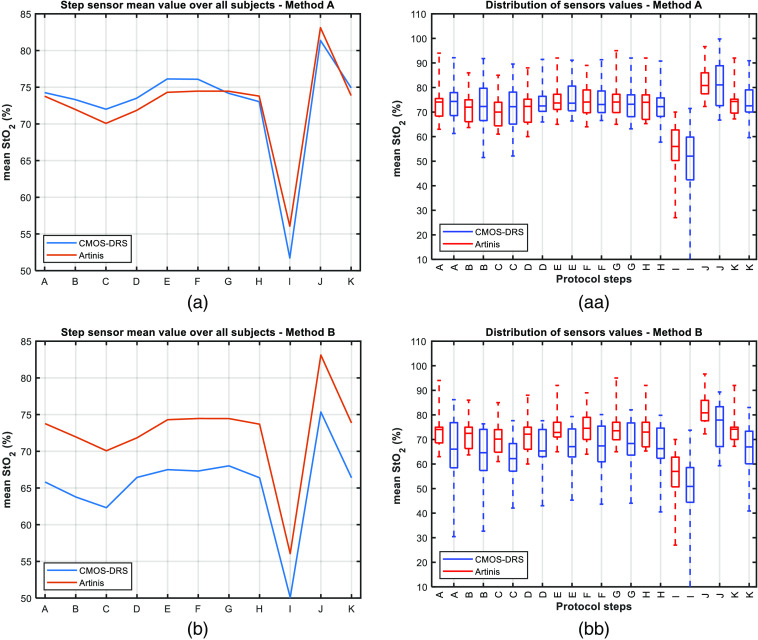
(a), (b) ${\mathrm{StO}}_{2}$ mean value for all subjects measured with the reference (red) and CMOS-DRS (blue) ${\mathrm{StO}}_{2}$ sensors. (aa), (bb) Value distribution for each step as measured with the reference (red) and CMOS-DRS sensor (blue). On each box, the median value is represented by the central mark. Bottom and top edges of the box indicate the 25th and 75th percentiles of the measurements, respectively. (a, aa) using method A. (b), (bb) using method B.

**Fig. 9:**
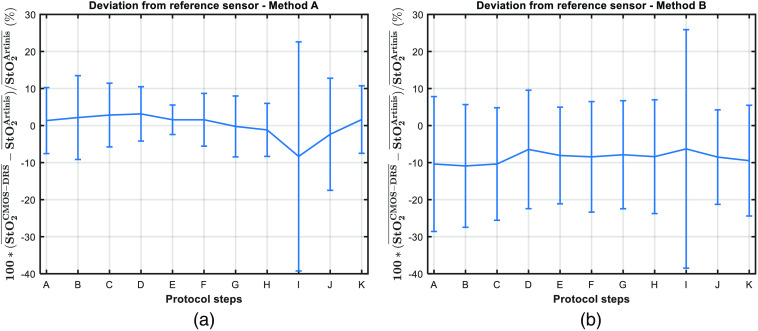
Mean deviation between ${\mathrm{StO}}_{2}$ values measured with the CMOS-DRS sensor and the Artinis. For each step, deviations are averaged over all subjects and expressed in percent of the expected value (Artinis measurement). Error bars correspond to the standard deviation of this mean deviation over all subjects. (a) Using method A. (b) Using method B.

We have completed experiments with 20 volunteers, among which 19 were exploitable. We present the results with an emphasis on one subject: subject 14 (Figs. 6 and 7). We present results for the entire study in Figs. 8 and 9. Tables of results for each subject at each step are given in appendix.

### 3.1 Optical Properties

As described in Sec. 2.3, the first step of data processing consists of extracting absorption and scattering properties from reflectance measurements. For most subjects (15), we used as reference PDMS phantom, phantom B1. For darker skin (4), we had to use phantom B2, which is more absorbent to avoid saturation in resulting tissue absorption spectra. With our calibration method, the properties are better estimated if the properties of the reference phantom are close to those of the subject.^15^

As an illustration, the optical coefficients $\mu_{a}$ and ${\mu_{s}}^{\prime }$ measured by the CMOS-DRS device on volunteer 14 at the three acquisition wavelengths are shown in Figs. 6(a) and 6(b), respectively. Letters on top of figure correspond to protocol steps as shown in Fig. 3.

### 3.2 Oxygen Saturation, Method A

The ${\mathrm{StO}}_{2}$ levels measured by the Artinis and the CMOS-DRS systems for volunteer 14, using method A are shown in Fig. 7(a). ${\mathrm{SpO}}_{2}$ readings recorded by the SenTec system on the left arm are also displayed. We observed that the ${\mathrm{SpO}}_{2}$ measurements are much more sensitive to desaturation (A, B, C) than ${\mathrm{StO}}_{2}$ readings. Changes in arterial blood versus tissue oxygenation in response to changes in inspiratory oxygen pressure can indeed show distinct patterns, as previously shown by our group.^27^^,^^28^ Tissue vascular (increase in regional blood flow or capillary recruitment) and metabolic (e.g., reduction in tissue metabolism in hypoxia) responses can lead to smaller reductions in local oxygen saturation compared with arterial oxygen saturation. Therefore, in the context of this study inducing hypoxic conditions by decreasing inspiratory oxygen pressure (which can be different from tissue hypoxia due to local ischemia for instance), this distinct pattern of ${\mathrm{SpO}}_{2}$ versus ${\mathrm{StO}}_{2}$ seems to be expected.

Conversely, the ischemia (steps I, J) has no effect on the ${\mathrm{SpO}}_{2}$ level as oxygenation changes are induced only locally by the cuff inflation. This confirms that the ${\mathrm{StO}}_{2}$ decrease detected by the Artinis and the CMOS-DRS sensor is exclusively induced by the cuff inflation. ${\mathrm{StO}}_{2}$ levels yielded by the CMOS-DRS device match the values obtained from reference measurements within 5%.

The StO2 levels measured by the Artinis and the CMOS-DRS systems were compared for all subjects, as for subject 14. To resume the comparison between the two sensors, we calculate representative figures over the whole subject’s panel. The ${\mathrm{StO}}_{2}$ evolution obtained from the CMOS-DRS and Artinis system averaged for each step, and averaged over all 19 subjects is shown in Fig. 8(a). In Fig. 8(aa), we plotted the ${\mathrm{StO}}_{2}$ value distribution for each step and for the two systems. On each box (red for reference sensor and blue for CMOS-DRS sensor), the central mark indicates the median, and the bottom and top edges of the box indicate the 25th and 75th percentiles of the measurements, respectively. The whiskers extend to the most extreme data points considered as outliers (Matlab© boxplot function). Results show that mean values are in accordance [Fig. 8(a)] and that the distribution of values is typically more extensive for the CMOS-DRS sensor [Fig. 8(aa)].

During desaturation steps, a slow decrease (up to 10% with the Artinis sensor for the subject of Fig. 7) was observed. With the CMOS-DRS sensor, the first hypoxia step (B) is not visible; a slight decrease is observed for the second hypoxia step (C).

The desaturation steps are slightly better detected in Fig. 8(a) with the Artinis reference sensor than with our CMOS-DRS sensor. However, the recovery to normal saturation (ambient and capnia steps) follows the same slope.

The ability of the CMOS-DRS sensor to monitor the decrease of tissue oxygenation during ischemia is shown in Figs. 7(a) and 8(a). Moreover, the slight rise in ${\mathrm{StO}}_{2}$ value, typically observed after cuff release,^2^ is also detected both for the CMOS-DRS device and reference sensor. The hypoxia decrease (I) is of the same order as for the Artinis sensor, the increase after recovery (J) is detected.

The deviation of CMOS-DRS mean values with respect to reference sensor step values over all subjects at each protocol step is shown in Fig. 9(a). The deviation to the Artinis reference sensor is under 5% for all protocol steps excluding the ischemia step, where the ${\mathrm{StO}}_{2}$ decrease is typically overestimated by 10%. The largest uncertainty lies on the first steps, we noted that our sensor requires a time of stabilization after installation on the skin. Furthermore, for the ischemia step, for two subjects, we observed an overestimation of the decrease of the ${\mathrm{StO}}_{2}$ reaching almost 0. This explains the huge error bar of step I.

### 3.3 Oxygen Saturation, Method B

We present in Fig. 7(b) the results of the ${\mathrm{StO}}_{2}$ calculations for volunteer 14, using method B, which is independent of an anterior calibration step using a reference sensor. For subject 14 shown in Fig. 7(b), the two hypoxia steps (B and C) are now visible in comparison with Fig. 7(a) where they were not. The Artinis values go from 75% (step A) to 69% (step C), whereas CMOS-DRS values go from 70% (step A) to 62% (step C).

For ischemia, the same observations as for the method A are made. The ischemia decrease (J) is of the same order as for the Artinis sensor from 75% (step I) to 49% (step J) for the Artinis and from 70% (step I) to 50% (step J) for the CMOS-DRS sensor. The increase after recovery (K) is well detected in both cases.

As for method A, we plotted in Figs. 8(b) and 8(bb) the mean ${\mathrm{StO}}_{2}$ value for all 19 subjects and value distribution after normalization for each step using method B for calculation. We observed a difference between Artinis and CMOS-DRS curves for each step. CMOS-DRS values are for all steps below Artinis ones, by an offset of almost 8%. However, desaturation and ischemia decreases follow the same slope.

We plotted in Fig. 9(b), the deviation to the reference sensor calculated for all subjects at each protocol step. Using method B, the deviation to the Artinis reference sensor is now around 10% for all protocol steps because of the negative offset. The greatest uncertainty lies again on the first steps and ischemia (for two subjects the decrease is overestimated).

## 4 Discussion

Despite the great accordance between our CMOS-DRS sensor and the Artinis, we observe discrepancies for certain subjects. The methods are quite dependent on the optical properties computation, the first step of data processing. This result is itself dependent on the calibration process, using a phantom of known optical properties as reference. For example, we have observed, for certain subjects, similar values for $\mu_{a}$ parameter but a cross-talk in ${\mu_{s}}^{\prime }$ parameter, values at 611 and 660 nm being inversed. This phenomenon suggests that the reference phantom was not adapted. In those cases, we adjusted the reference phantom, using B1 for lighter skin and B2 for darker ones.

One limitation identified and discussed in the first publication^7^ was the choice of wavelengths. We recommend for a second future version of the CMOS-DRS sensor to use an LED in the near-infrared range (around 940 nm as in most oxymeters) instead of 611 nm. Actually in the range 590 to 640 nm, the absorption of oxyhemoglobin varies very rapidly and the measurement is therefore highly sensitive to the chosen wavelength. The distribution of values, typically more extensive for the CMOS-DRS sensor, may be related to uncertainties in the optical properties determination prior to ${\mathrm{StO}}_{2}$ computations.

A first analysis after the acquisition on the first six subjects showed that our sensor requires a time of stabilization after installation on the skin, estimated to 5 min. This is clearly visible on subject 14 shown in Fig. 7(a). For the first hypoxia level (step B), we can see the ${\mathrm{StO}}_{2}$ value goes on increasing and starting to decrease only upon second hypoxia level (step C), whereas for the reference Artinis sensor the hypoxia is clearly visible for the two steps B and C. This may be due to a slightly local heating of the skin by the LEDs. Note that the Artinis system was turned on immediately upon installation on the arm and prior to the CMOS-DRS device, which was turned on only at the beginning of step A. To confirm this hypothesis, we started CMOS-DRS measurements prior to the first step A for the two last subjects of the trial, for which the ${\mathrm{StO}}_{2}$ decrease was detected at step B. Note that method B, which uses the green acquisition to learn the “other” absorption coefficient, allows compensation for this stabilization time, by correcting the signal through a time dependent $\mu_{a}^{\text{other}}$ parameter. For subject 14, as shown in Fig. 7(b), the two hypoxia steps (B and C) are then visible. In this trial, we generate hypoxia and ischemia over a short enough time to test first the sensitivity of the system and second the capacity of a short acquisition time. For continuous monitoring (of the order of a few seconds), we are effectively limited by this stabilization time. In the monitoring of oxygenation of the flaps, thrombosis can occur quite “suddenly” but the order of magnitude is more like 1 h.^1^^,^^3^ In this case, an acquisition every 5 min might be sufficient and stabilization time is less of a problem.

Another difference with the Artinis reference sensor is the overestimation of ischemia. For 9/19 subjects, the CMOS-DRS sensor overestimates the ${\mathrm{StO}}_{2}$ decrease as it can be seen on mean values of Figs. 8(a) and 8(b). However, it is generally in accordance with the Artinis for the increase after recovery. We can remark that method B allows the signals to be readjusted in a given oxygenation state, yet a negative offset of almost 8% is observed. This difference in absolute values may be explained by the fact that the two sensors do not probe the tissue at the same depths. The NIRS has a measurement depth of about half the distance between the transmitter and the receiver (which is in our case ∼2  cm), meaning that both the dermis and muscles can be probed. Conversely, the CMOS DRS was designed to probe the dermis only, i.e., limited in depth to a few millimeters.

This work shows that the CMOS-DRS is a system allowing qualitative continuous monitoring of ${\mathrm{StO}}_{2}$. In addition, the use of the third wavelength (method B) makes our system autonomous and independent of a reference system. However, the discrepancies observed between quantitative measurements of ${\mathrm{StO}}_{2}$ between CMOS-DRS system and PortaLite Artinis reference system may not entirely explained by the different probing depths of devices. Further studies need to be engaged to fully assess the ability of CMOS-DRS approach to provide quantitative measurements. In particular, a complementary study involving oxygenated and deoxygenated hemoglobin phantoms with data on a larger spectral range should be conducted. The experiments presented in this paper, however, demonstrate that our approach is suitable for qualitative detection of ischemia, showing potential for the detection of free flaps failure in postoperative monitoring.

## 5 Conclusion

This clinical trial that aimed at testing two sensors for the monitoring of ${\mathrm{PtCO}}_{2}$ and ${\mathrm{StO}}_{2}$ allowed us to investigate their implementation on the same platform.^21^ Regarding the CMOS-DRS sensor, this clinical trial and the comparison with reference sensors helped us to understand the $\mu_{a}$ estimation discrepancy previously seen.^7^ Results obtained assess the potential of the system for *in vivo* tissue oxygenation monitoring in clinical settings.

In the future, further work has to be done toward the development of a fully integrated sensor. This will notably involve the development of an embedded software for the online computation of the ${\mathrm{StO}}_{2}$ and eventually the use of Bluetooth technology to suppress the USB connection.

Besides, this work has been done with the underlying objective of layered media analysis. A theoretical validation of the approach has already been done through simulations of multilayer propagation with both vertical and angled sources.^8^ In particular, an angled source or detector incidence enhances the sensitivity to shallow depths. For this purpose, a fourth source having 35-deg incidence angle has been implemented in the device for experimental verification. Further studies will be carried out to validate the approach, which may lead to improvements in measurements of optical parameters in the dermis independently of the epidermis.

In summary, a CMOS-based contact imaging system for srDR measurements has been developed. This first proof-of-concept has led to the development of a low-cost, wearable prototype designed for the monitoring of tissue oxygenation *in vivo*. The instrument was evaluated on tissue-simulating phantoms in comparison with SFDI in a preliminary work^7^ and to NIRS systems in this study. The preliminary study assessed the capacity of our approach for the qualitative *in vivo* monitoring of tissue oxygenation, whereas the clinical trial demonstrates a real prospect for quantitative results. In particular, our approach based on a three wavelengths acquisition shows great potential for quantitative results independently of any calibration reference device.

Others advantages of the CMOS-DRS technique over the “noncontact imaging” modality are low cost, small footprint, a reduced sensitivity to movement, an easier positioning of the system on the patient that make it easier to use for nursing staff.

Future work will address further validation for quantitative results, the integration aspect of the system, through software embedment and implementation of wireless data transfer, making eventually a low cost, easy-of-use system for continuous ${\mathrm{StO}}_{2}$ monitoring. The characterization work will be continued to make the measurement quantitative and allow in the future the use of the CMOS-DRS system for the monitoring of flaps and the detection of flap failure. Moreover, the coupling with the measurement of ${\mathrm{ptCO}}_{2}$ on the same platform may be a real advantage in the monitoring of respiratory pathologies such as sleep apnea for example.

## 6 Appendix

The ${\mathrm{StO}}_{2}$ results for each subject at each protocol step, are given in Tables 4–6 for respectively, the CMOS-DRS method A, CMOS-DRS method B, and the reference device Artinis PortaLite. In these tables, ${\mathrm{StO}}_{2}$ values are calculated by taking the average over the last 30 s of each step; MD stands for missing data.

**Table 4:** ${\mathrm{StO}}_{2}$ (%) for each subject at each step. CMOS-DRS method A.

Subject	A	B	C	D	E	F	G	H	I	J	K
01	63.7	51.5	69.9	69.2	70.1	72.9	72.8	75.7	52.0	82.8	76.4
02	68.6	65.7	64.6	66.5	66.9	66.8	66.6	68.0	63.5	69.3	66.6
03	72.1	84.3	71.1	71.2	81.2	66.8	65.6	61.0	42.7	91.6	90.5
04	78.7	87.4	77.9	72.5	73.6	71.4	71.5	72.2	58.6	99.8	72.0
05	74.9	72.3	70.8	72.3	70.6	70.2	72.6	69.9	57.9	72.8	71.4
06	68.4	66.7	65.2	67.7	68.4	68.6	68.6	68.3	55.0	68.5	67.9
07	66.1	67.9	73.9	74.8	72.9	74.3	77.2	73.8	71.5	88.1	78.4
08	77.7	77.0	75.8	77.4	77.2	77.3	77.8	77.3	67.3	77.9	77.5
09	75.1	74.4	79.6	76.3	80.5	77.4	75.8	75.5	**0.0**	81.1	72.7
10	76.0	71.9	64.9	72.0	72.0	73.1	63.2	72.4	40.7	74.5	72.3
11	61.3	58.5	57.0	66.0	66.4	66.6	66.4	66.7	47.6	66.8	59.6
12	74.3	73.1	72.2	72.6	72.8	72.7	73.2	72.0	66.3	72.8	72.5
13	92.1	91.8	89.6	91.4	91.1	91.3	92.0	90.8	44.7	91.5	90.9
14	85.2	88.4	78.7	76.7	82.7	87.8	85.6	83.1	41.5	90.6	81.0
15	69.9	66.1	52.1	75.2	80.2	82.1	76.4	57.8	34.0	72.0	70.7
16	**MD**	72.2	76.7	72.9	81.4	79.5	79.3	82.3	71.9	87.2	73.0
17	73.2	78.2	79.7	77.0	74.6	75.6	76.0	72.0	53.8	81.3	65.3
18	**MD**	**MD**	**MD**	**MD**	**MD**	**MD**	**MD**	**MD**	**MD**	**MD**	**MD**
19	91.6	69.6	73.8	70.6	80.8	84.2	76.9	76.0	51.6	88.3	86.7
20	67.7	76.1	74.5	74.0	83.0	87.1	71.7	**MD**	62.1	89.4	78.1

**Table 5:** ${\mathrm{StO}}_{2}$ (%) for each subject at each step. CMOS-DRS method B.

Subject	A	B	C	D	E	F	G	H	I	J	K
01	77.3	76.0	66.3	74.4	74.3	78.2	78.3	74.0	73.8	89.3	83.0
02	77.6	71.7	68.4	72.6	73.6	73.6	72.1	74.6	63.5	81.0	70.2
03	79.5	74.1	77.7	77.6	77.4	75.4	80.0	79.8	42.4	81.9	62.1
04	30.4	32.7	42.1	43.0	45.3	43.7	44.1	40.5	6.1	59.3	40.9
05	61.8	55.5	54.2	65.6	65.0	65.7	65.7	63.1	51.4	67.1	60.1
06	50.7	49.2	45.8	52.5	57.2	56.9	54.5	56.7	36.6	63.4	58.9
07	57.4	58.0	57.1	58.1	65.5	60.5	55.7	56.3	46.1	61.2	55.6
08	86.2	76.3	71.7	76.8	79.3	80.1	76.7	78.3	48.7	84.0	76.4
09	67.5	66.9	55.7	64.5	61.7	66.6	67.8	62.3	50.4	86.3	67.3
10	76.9	76.4	76.0	77.2	74.1	73.4	82.1	72.9	58.3	84.2	73.4
11	58.3	56.9	61.4	63.0	64.3	64.5	64.5	66.3	60.9	77.3	66.1
12	62.7	61.1	61.3	65.0	66.0	66.7	66.3	65.8	44.4	69.4	66.9
13	70.5	65.5	62.6	64.0	68.2	68.1	68.9	66.3	58.6	79.3	67.0
14	66.8	65.0	65.3	69.4	68.8	68.7	70.5	68.8	53.6	75.5	68.3
15	74.6	74.8	68.3	74.1	77.1	78.1	78.5	75.2	35.5	78.5	77.2
16	**MD**	67.1	67.1	67.4	62.3	63.1	66.3	59.9	55.2	72.6	68.4
17	65.3	63.0	61.8	73.7	78.0	78.4	74.1	75.9	57.2	83.3	76.1
18	**MD**	**MD**	**MD**	**MD**	**MD**	**MD**	**MD**	**MD**	**MD**	**MD**	**MD**
19	58.5	64.2	62.7	64.4	62.9	60.9	62.5	65.6	59.2	71.1	60.1
20	62.8	57.4	58.6	58.9	61.6	56.4	63.6	59.2	49.6	67.1	63.7

**Table 6:** ${\mathrm{StO}}_{2}$ (%) for each subject at each step. Artinis PortaLite.

Subject	A	B	C	D	E	F	G	H	I	J	K
01	67.6	65.4	63.2	63.2	68.4	66.7	66.9	66.9	50.7	72.3	68
02	66.8	63.7	62.2	65.4	65.9	66.7	69.5	65.3	51.4	77.0	67.2
03	74.1	73.6	70.3	74	76	75.5	76.4	74	62.7	80.8	74.3
04	71.3	70.1	69.7	71.5	71.7	73.2	72.7	70.7	67.9	75	71.3
05	72.8	71.3	70	71.4	71.9	71.8	71.2	71	62.8	76.4	70.5
06	68.6	66.3	64.8	66.1	69.2	68.9	69.9	66.2	51.8	77.6	68.3
07	74.7	72	68.2	72.3	73.7	74.1	74.1	75.1	61.8	80.8	74.8
08	75	75	74	76	79	79	76	78	70	79.6	74
09	82	75	75	78	80	79	78	80	45	85.7	75
10	75	75	71	75	76	80	80	74	58	85.9	76
11	63	65	61	66	65	64	65	67	43	85.1	68
12	66	64	62	64	72	70	69	71	49	94.5	75
13	94	86	85	88	92	89	95	92	56	96.7	92
14	77	74	72	74	78	78	76	77	53	86.3	75
15	75	70	65	60	72	70	70	67	27	94.47	70
16	**MD**	78	77	77	78	79	77	80	65	86.41	79
17	77	77	75	77	75	76	78	76	61	84.78	77
18	**MD**	**MD**	**MD**	**MD**	**MD**	**MD**	**MD**	**MD**	**MD**	**MD**	**MD**
19	74	73	74	74	77	79	77	77	65	80.27	76
20	74	73	72	72	71	75	73	**MD**	64	79.56	72
